# Changes in Major Global River Discharges Directed into the Ocean

**DOI:** 10.3390/ijerph16081469

**Published:** 2019-04-25

**Authors:** Xiaoqing Shi, Tianling Qin, Hanjiang Nie, Baisha Weng, Shan He

**Affiliations:** 1 State Key Laboratory of Simulation and Regulation of Water Cycle in River Basin, China Institute of Water Resources and Hydropower Research, Beijing 100038, China; sxq18@mails.tsinghua.edu.cn (X.S.); nhj16@mails.tsinghua.edu.cn (H.N.); wengbs@iwhr.com (B.W.); hsiwhr61@163.com (S.H.); 2 Department of Hydraulic Engineering, Tsinghua University, Beijing 100084, China

**Keywords:** river discharges, trend, spatial distribution, climate change, human activities

## Abstract

Under the influence of global climate change, the discharges of major global rivers directed into the ocean have undergone significant changes. To study the trends and causes in discharge variation, we selected 40 large rivers and analyzed their annual discharges near their estuaries from 1960 to 2010. The method of runoff variation attribution analysis based on the Budyko hypothesis for large-scale basins was developed, in which influencing factors of human activities and glacial melting factors were added to the formula. The contribution rate of climate factors and human activities to changes in discharge were quantitatively identified. Climatic factors include precipitation, evapotranspiration and glacial melting. Human activity factors include underlying surface and artificial water transfer. The contribution rate is determined by the elastic coefficient, which is obtained by the ratio of change rate of each factor and the change rate of runoff. The results indicated that the discharges predominantly showed downward trends with a few upward trends. Rivers in North America and Africa showed downward trends, and those in Europe principally showed upward trends. Climate was the main influencing factor of discharges changes, and only approximately 25% of river discharges were greatly affected by human activities. River discharges in 75% of the basins which mainly contains subtropical monsoon humid climate and savanna climate zones showed upward trends. In the four basins which are mainly contains tropical rainforest climate and tropical monsoon climate, they all showed downward trends. The trend of discharges in the temperate monsoon climate, temperate continental climate, and temperate maritime climate cannot be accurately judged because of irregular variation. The discharges in the mid-high latitudinal zones predominantly showed upward trends, while those in the mid-low latitudinal zones with the influence of human activities showed downward trends.

## 1. Introduction

The discharge of large rivers into the oceans constitutes an important component of the global hydrological cycle and an important link for the coupling between terrestrial and oceanic hydrological cycles. In recent years, the global mean surface temperature (GMST) has continued to increase [1,2]. The frequency of extreme events such as heavy rainfall and drought has increased significantly in various regions of the world [2,3,4,5,6]. Combined with the impact of human activities, discharges may change significantly [7,8]. At present, the environmental degradation of many estuaries in the world is a serious issue that poses a threat to water supply security, ecological environment safety, and sustainable socioeconomic development. Discharge directed into the ocean represents the final process in the complex water cycle, and its changes are comprehensive manifestations of climate change and human activities. Therefore, it is necessary to study the variational characteristics and causes of discharge directed into the oceans. Understanding global river discharge can also be helpful for exploring the evolution of the global water cycle and providing a scientific basis for mitigating the global water crisis.

Many scholars have performed relevant research on the trend and causes of runoff in individual or several river basins. In the Arctic rivers, most studies show that the runoff has an upward trend and the changes are more affected by climatic factors. The runoff of the Yenisei River showed a significant upward trend in 1936–1998, with a downward trend in precipitation, which was most likely due to the regulation of the reservoir and dam [9,10]. The runoff of the Lena River has an upward trend in 1936–2001, but the precipitation has only a slight upward trend, which was not enough to prove that the runoff change was related to precipitation, so maybe it was due to the extended flood season during the second half of the last century. From the 1940s to the 21st century, the trend of runoff in the Ob and Yukon Rivers were not obvious [9,11,12], but the average runoff of six Arctic rivers (Northern Dvina, Pechora, Ob, Yenisei, Lena, and Kolyma) increased by 7% [13,14]. There are many reasons for the change in runoff in Arctic rivers, including changes in precipitation and snowmelt, permafrost degradation, and increased fire frequency [12,15].

In the other rivers, the trend of runoff changes was different and it may be affected by both climatic factors and human activities. Since the 1940s, the runoff in the Mississippi River Basin has increased with an increase in precipitation, changes in land use/cover and vegetation cover has affected the hydrological cycle of the basin [16]. In the Columbia River Basin, the original flow decreased by 16.5% since 1858, of which 8–9% was attributed to climate change and 7–8% to irrigation use [17]. Since the 1960s, the annual runoff in the Yangtze River Basin showed an insignificant upward trend [18], and the runoff, precipitation, and evapotranspiration were increased in the St. Lawrence River Basin [19]. In the Yellow, Colorado, Murray-Darling, and Orange River Basin, the runoff showed a downward trend, and the reasons for the reduction include both climate change and human activities such as excessive water abstraction for irrigation and industrial production [18,20,21,22,23]. But different studies had different conclusions about the influencing factors. The low runoff in the Murray-Darling River Basin was attributed to the arid climate, which contributed 71% to the measured runoff reduction in 1997–2006 [20]. In the Yellow River Basin, Piao et al. [18] concluded that although the impact of human activities was significant, climate change was still dominant, while Kong et al. [21] stated that the contribution rates of water abstraction and precipitation were 42.2% and 39.2%, respectively.

Some scholars have studied global major river runoff changes. Pekárová et al. [24] studied the temporal and spatial variation characteristics during the 19th and 20th centuries. The results showed that river runoff changes were not obvious in Europe. Rivers in northeastern or southeastern Europe had similar periods of drought. The Siberian Rivers in northern Asia had different extreme times. Dai et al. [25] selected runoff data from 925 rivers from 1948–2004 for analysis and found that only approximately one-third of the rivers (e.g., the Congo River and the Mississippi River) show statistically significant trends. It was believed that during the study period, the impact of human activities on global major river runoff was much less than climate change. Tang and Lettenmaier [26] selected 194 large rivers around the world, covering 72% of the world’s land area (excluding Antarctica and Greenland), and analyzed the sensitivity of runoff. The results showed that under various emission scenarios, the sensitivity of runoff to climate change was relatively stable. In most parts of the world, runoff changes due to climate variables were generally linearly related to global mean temperature changes.

The attribution analysis methods of runoff change mainly include hydrological model and water balance model [27]. The water balance model based on the Budyko hypothesis is often used to analyze the contribution of climate characteristics and underlying surface features to runoff variation [27,28,29,30,31,32]. Wang et al. [32] considered the effects of frozen ground degradation on runoff in the source region of the Yellow River. Ning et al. [31] studied the effects of vegetation dynamics and climate seasonality on water balance in the Loess Plateau.

When analyzing the changes of discharge in some large rivers, most of the previous studies select only one or several river basins, and the time series of data are different, which makes it difficult to compare runoff changes in multiple basins in the same time period. Method of attributive analysis of runoff variation based on the Budyko hypothesis is rarely used in the analysis of runoff variation at large basin, it is mostly used to study the source region of rivers. In this research, the collected data are interpolated to the same time series by using various methods, and the processed runoff data is analyzed to obtain the variation and influencing factors in the major river basins in the same period, which will provide a basis for studying changes in basin runoff and water cycle over a certain period.

## 2. Materials and Methods

### 2.1. Study Area

**Figure 1:**
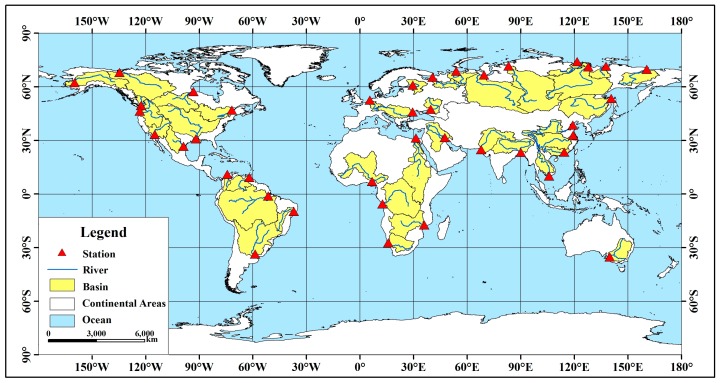
Study area.

This study considers factors such as data richness, geographical representation, natural and social development of the basin and the extent of the basin area. Forty major rivers in the world are selected, in Asia, Africa, North America, South America, Europe, and Oceania and cover a wide range of climatic zones and latitudes. The drainage area, flow regions, and natural conditions are different. The total drainage area is approximately 60 × 10^6^ km^2^, accounting for approximately 45% of the global land area. The distribution of the selected river basins is shown in Figure 1.

### 2.2. Data

The discharge data used in this paper is collected from the Global Runoff Data Center (GRDC), the U.S. Geological Survey (USGS) Hydro-Climatic Data Network (HCDN), the Canada HYDAT Database (HYDAT), the Australian Bureau of Meteorology (ABM), the South American Observation Service SO HYBAM (formerly the Environmental Research Observatory) (http://www.ore-hybam.org/), and other research projects. Considering the representativeness of the geographical locations of the sites and the reliability and completeness of the data, we selected the observed and simulated annual discharge data for the major rivers at the stations near the estuaries for the past 50 years (1960-2010) and then processed the data through interpolation and prolongation to make a complete series. The data sources and references are shown in Table 1.

The global digital elevation (DEM) data used in this study are derived from ASTER GDEM data with a resolution of 30 m × 30 m. Considering that the coverage area of the studied basins is large, we resampled the DEM data at a resolution of 1 km × 1 km to generate the river basins in ArcGIS.

The precipitation and temperature data are monthly data from 1900 to 2014 with a resolution of 0.5° × 0.5°, which was derived from the Global Precipitation Climatology Center (GPCC). On this basis, the monthly precipitation and temperature raster data of 40 watersheds from 1960 to 2010 were extracted, and their annual values were calculated.

The actual evapotranspiration and potential evapotranspiration data from 1948 to 2010 with a monthly scale and a resolution of 0.25° × 0.25° were derived from the Global Land Surface Data Assimilation System (GLDAS). According to the research, the monthly data of actual evapotranspiration and potential evapotranspiration from 1960 to 2010 in 40 watersheds -were extracted, and the annual values were calculated.

### 2.3. Methods

#### 2.3.1. Linear Tendency Estimation

In this method, *x*_i_ represents a measured variable for a total number of samples *n*, and *t*_i_ represents the time corresponding to *x*_i_. A linear regression equation can be established between *x*_i_ and *t_i_*:

(1) $$\hat{x_{i}}{=a+bt}_{i}\;\left(i=1,2,\ldots ,n\right)$$

where *a* is the regression constant and *b* is the regression coefficient. The sign of b indicates the trend tendency of the climate variable *x*.

#### 2.3.2. Mann-Kendall Test

A Mann-Kendall (hereinafter referred to as M-K) test is a nonparametric statistical testing method that can be used to analyze the trend and mutation of a time series. In an M-K trend test [49], the statistical value Z is defined as:

(2) $$Z=\{\begin{array}{cc} \frac{\left(S-1\right)}{\sqrt{\frac{n(n-1)(2n+5)}{18}}} & S>0\\ 0 & S=0\\ \frac{\left(S+1\right)}{\sqrt{\frac{n(n-1)(2n+5)}{18}}} & S<0 \end{array}$$

where *n* is the length of the sequence, S is a statistical value:

(3) $$S=\overset{n}{\underset{i=2}{\sum }}\overset{i-1}{\underset{j=1}{\sum }}{\mathrm{sgn}(X}_{i}{-X}_{j})$$

where sgn() is a symbolic function, when (*X*_i_ − *X*_j_) is less than, equal to, or greater than zero, sgn(*X*_i_ − *X*_j_) is −1, 0, or 1 respectively.

For a random sequence *X* = (*x*_1_, *x*_2_, …, *x*_n_), at the given significance level *α*, the critical test value *Z_α/2_* can be found from a normal distribution table. Then, |Z| and *Z_α/2_* are compared to determine the significance of the change.

#### 2.3.3. Cumulative Departure Curve

The cumulative departure curve is a commonly used method for judging the trend change of a curve. For a sequence *x*, the cumulative departure at time t is expressed as follows:

(4) $$\hat{x_{t}}=\overset{t}{\underset{i=1}{\sum }}\left(x_{i}-\bar{x}\right)\;\left(t=1,2,\ldots ,n\right)$$

where:

(5) $$\bar{x}=\frac{1}{n}\overset{n}{\underset{i=1}{\sum }}x_{i}$$

All of the cumulative distances are calculated, after which we can plot the cumulative departure curve to conduct trend analysis.

#### 2.3.4. Method of Runoff Variation Attribution Analysis Based on the Budyko Hypothesis for Large-Scale Basins

The water balance mechanism in the natural water system is the amount of input water equal to the amount of output water plus the change in the water storage capacity. The formula is expressed as:

(6) $$W_{\mathrm{input}}-W_{\mathrm{output}}=\pm \Delta W$$

Further expressed as:

(7) R=P−E±ΔS

where R represents the observed runoff in the natural system, that is, the natural runoff; P represents the precipitation; E represents the actual evapotranspiration; ∆S represents the change in the storage capacity.

It is generally considered that in the natural state, when the large basin scale is studied and the research period is long, the change in the water storage capacity can be neglected, that is, ∆S = 0. The expression is as follows:

R = P − E(8)

The coupled water-energy balance based on the Budyko hypothesis and named the Choudhury-Yang formula [50] characterizes the long-term water-energy balance relationship of the basin under certain climatic and underlying conditions. There are two preconditions for applying this formula. One is that the basin must be a closed basin, that is, the surface and the underground watershed are consistent; the second is that the change in the water storage capacity of the basin for many years is negligible [29]. The concrete expression is as follows [51]:

(9) $$E=\frac{{\mathrm{PE}}_{0}}{{{(P}^{n}{+E}_{0}{}^{n})}^{\frac{1}{n}}}$$

where E is the average annual actual evapotranspiration (mm), P is the average annual precipitation (mm), E_0_ is the average annual potential evapotranspiration (mm), and *n* is the underlying surface coefficient, which can reflect the characteristics of the underlying surface of the watershed and can be calculated from the known parameters in the formula.

If we consider the impact of human activities on the water balance of the water system, then equation (8) can be written as:

(10) $$R_{s}{+R}_{r}=P-E$$

where R_s_ is the measured runoff and Rr is the amount of artificial water withdrawal and water transfer or glacial meltwater caused by global warming. When R_r_ indicates the amount of artificial water withdrawal, it is negative; when Rr indicates the amount of artificial water transfer or glacial meltwater, it is positive. P is the precipitation, and E is the actual evapotranspiration.

Then, bringing formula (9) into formula (10), the following expression is obtained:

(11) $$R_{s}{=P-R}_{r}-\frac{{\mathrm{PE}}_{0}}{{{(P}^{n}{+E}_{0}{}^{n})}^{\frac{1}{n}}}$$

From formula (11), the measured runoff R_s_ can be expressed as a function of P, R_r_, E_0_ and *n*, that is, R_s_ = *f* (*P*, *R_r_*, *E_0_*, *n*). The elastic coefficient refers to the ratio of the growth rate of two interconnected indicators in a certain period of time. For example, the precipitation elastic coefficient refers to the change in the runoff caused by the change in precipitation, expressed as:

(12) $$\varepsilon_{p}=\frac{{\mathrm{dR}}_{s}{/R}_{s}}{\mathrm{dP}/P}$$

Similarly $\varepsilon_{E_{0}}$, $\varepsilon_{R_{r}}$ and $\varepsilon_{n}$ can be calculated; thus, the total differentiation form of the runoff variation is obtained as follows:

(13) $$\frac{{\mathrm{dR}}_{s}}{R_{s}}{=\varepsilon }_{P}\frac{\mathrm{dP}}{P}{+\varepsilon }_{R_{r}}\frac{{\mathrm{dR}}_{r}}{R_{r}}{+\varepsilon }_{E_{0}}\frac{{\mathrm{dE}}_{0}}{E_{0}}{+\varepsilon }_{n}\frac{\mathrm{dn}}{n}$$

## 3. Results

### 3.1. Trends

**Figure 2:**
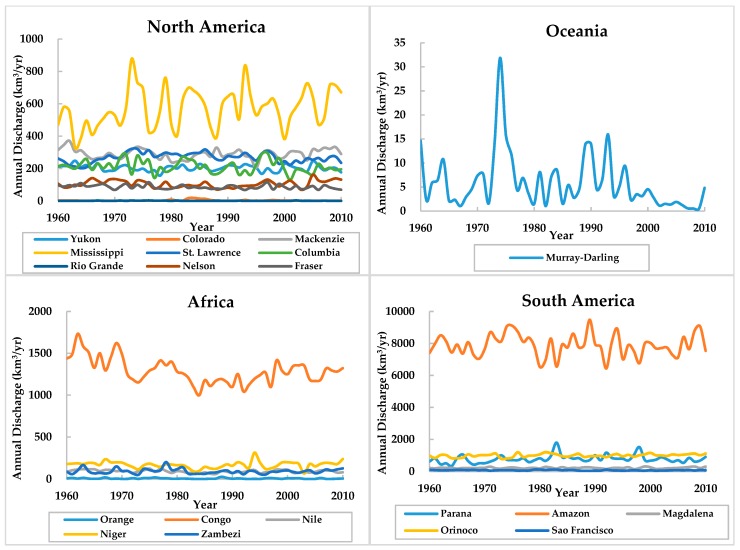

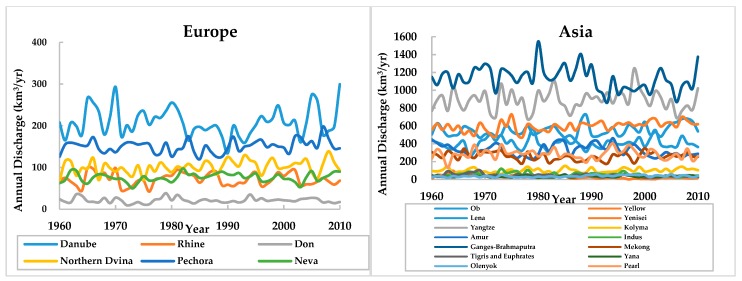
River discharge trends.

Through the annual discharge variation lines and M-K trend analysis in the 40 rivers from 1960 to 2010 (Figure 2, Table 2), the Mississippi River, Orinoco River, and five other rivers reached a significance level of α = 0.05 with a significant upward trend. Ten rivers, such as the Rio Grande and Murray-Darling Rivers, reached a significance level of α = 0.05 with a significant downward trend. The Columbia River and the Sao Francisco River reached a significance level of α = 0.1 with a significant downward trend. The trends in the other rivers did not pass the significance test of α = 0.1, indicating that their trends are not obvious. According to the comprehensive analysis, the discharges of 7 rivers increased significantly, and those of 12 rivers significantly decreased. Eleven of the other 21 rivers showed upward trends, and 10 showed downward trends, but their trends were not significant.

Among the nine selected rivers in North America, six rivers (two-thirds of the North American rivers) showed downward trends. Similarly, four of the six rivers (a large proportion) in Europe showed an upward trend. In Africa, one of the five selected rivers showed an upward trend, and thus, downward trends accounted for 80% of the total. In South America and Asia, the proportion of upward and downward trends was generally consistent.

Through the cumulative departure curve and M-K mutation test methods, the results for the segment trends were different. The results obtained by a comprehensive analysis of the two methods are shown in Table 3. Among the 40 rivers, there are 16 rivers showing the trend of “wet-dry”, 13 rivers showed the trend of “dry-wet”, eight rivers showed the trend of “dry-wet-dry”, and one river showed the trend of “wet-dry-wet”. The two rivers showed a trend of “wet-dry-wet-dry”, were the Pearl and Rhine rivers, but the mutation time of the Pearl River relatively lagged behind the Rhine River. Among them, the North American rivers mainly showed the trend of “wet-dry” with a mutation time around the 1980s; the five rivers in Africa all showed the trend of “wet-dry” with a mutation time around the 1970s; the mutation time of the European and Asian rivers was mainly in the 1980s and 1990s. The mutation time varies among rivers in South America.

### 3.2. Spatial Distribution

**Figure 3:**
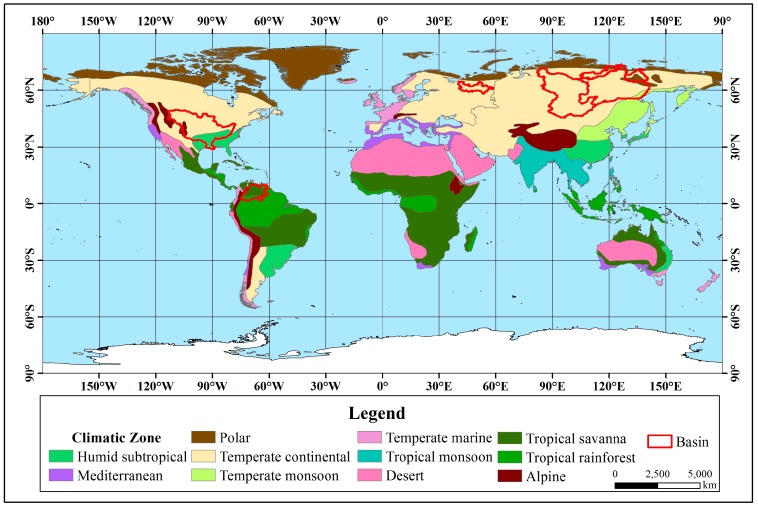
Distribution with a significant upward trend in river discharge.

From the statistical results, in the seven rivers with significant upward trends (Figure 3), the Orinoco River Basin is located in the tropics between 0° and 10°N, and the main climate type is a tropical savanna climate. The main climate in the other six river basins between 30°N–75°N, which is the mid-high latitude zone, is a temperate continental climate. Except for the Mississippi and Orinoco, the other five rivers belong to the Arctic river and are directed into the Arctic Ocean.

**Figure 4:**
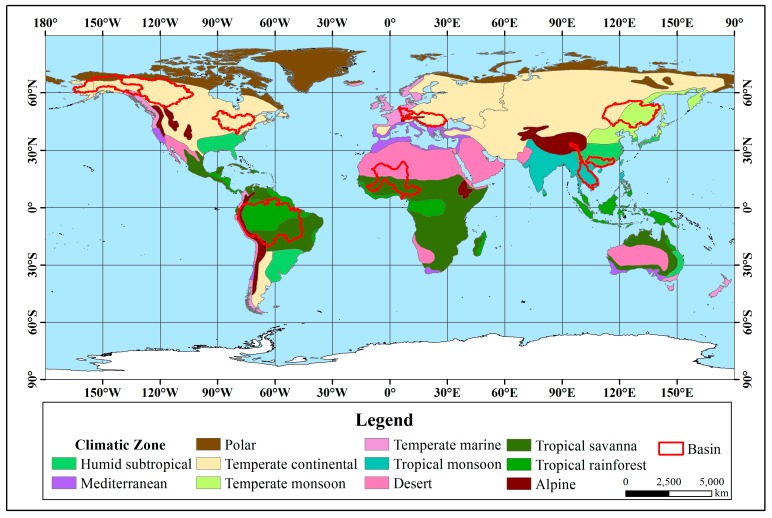
Distribution with a significant downward trend in river discharge.

The twelve rivers that showed significant downward trends (Figure 4) are all located in the mid-low latitude zone. The Congo, Nile and Sao Francisco rivers are located in tropical climates, and the other nine rivers are located in temperate climates. Regarding the main climate types, the Sao Francisco River Basin is located in a tropical savanna climate, and the rest have diverse climate types.

**Figure 5:**
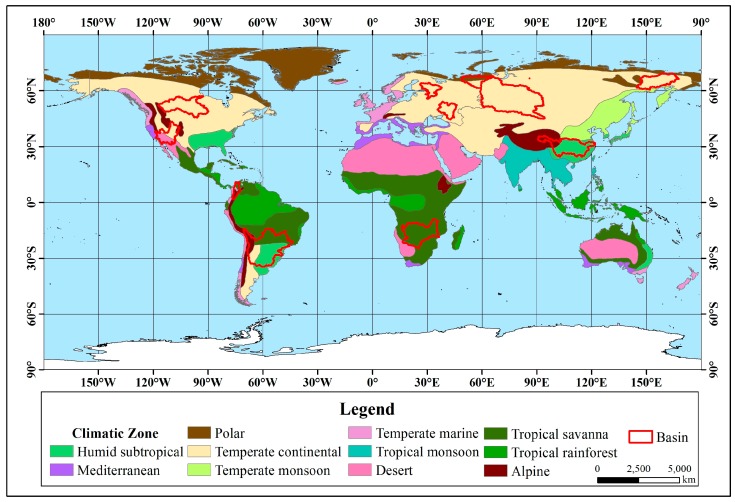
Distribution with an insignificant upward trend in river discharge.

Among the 11 rivers with an insignificant upward trend, from the statistical results (Figure 5), except for the Magdalena, Zambezi and La Plata-Parana River Basin, the remainder are located in the mid-high latitude zone. There are 6 basins located entirely in the temperate zone, and 8 basins are located in temperate continental climates.

**Figure 6:**
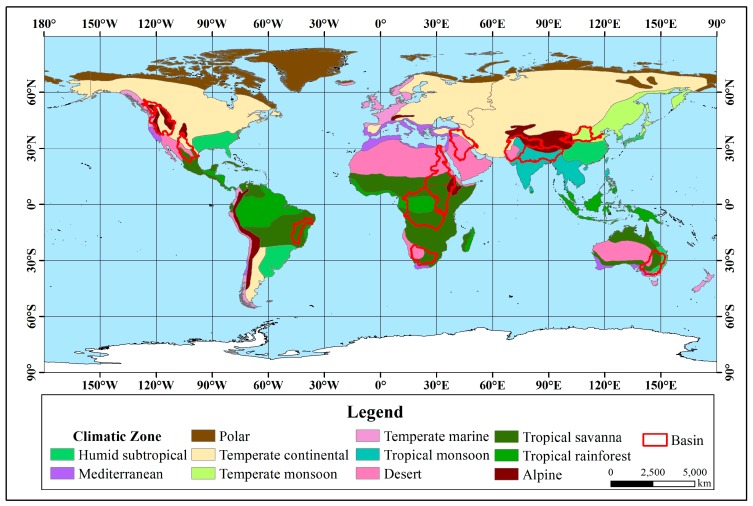
Distribution with an insignificant downward trend in river discharge.

Among the 10 rivers with an insignificant downward trend, from the statistical results (Figure 6), except for the Niger, Amazon, Mekong and Pearl River Basins located at low latitudes, the other six basins are located at the mid-high latitudes. The Niger and the Amazon River Basin are located in the tropics, the Mekong River Basin spans the tropics and the temperate zone, and the remaining 7 basins are located in the temperate zone. There are many climate types in the 10 basins.

### 3.3. Influencing Factors

In this study, the factors affecting climate change mainly include precipitation, evapotranspiration and glacial meltwater. The comprehensive contribution rate of precipitation and evapotranspiration is recorded as $\delta_{z}$, and the total contribution rate of climatic factors is recorded as $\delta_{c}$. The influencing factors of human activities mainly include underlying surface conditions and artificial water withdrawal and transfer, and the contribution rate is recorded as $\delta_{l}$. The underlying surface coefficient *n*, the elastic coefficient *ε*, and the contribution rate δ are shown in Table 4.

## 4. Discussion

### 4.1. Contribution Rate of Influencing Factors

From the above analysis results, we found that the contribution rate of precipitation in all basins is above 29%, and the precipitation elastic coefficient in 27 basins is larger than other factors. We can conclude that precipitation factors dominate the long-term discharge changes in these basins. The contribution rate of E_0_ is below 24%, and the impact is relatively small. The contribution rate of the R_r_ is less than 20%, except for the Yana River and the Lena River, indicating that the influence of this factor is relatively small.

In the seven basins where the discharge has a significant upward trend (Table 4), the $\delta_{z}$ values in the Lena River and the Yana River are less than 50%. The top three basins have precipitation contribution rates of 55%, 48%, and 42%, and the precipitation in these three basins has an upward trend. The Northern Dvina River has passed the 90% significance test; therefore, the discharge changes in these three basins are mainly attributed to the increase in precipitation. The precipitation trends of the Olenyok, Yenisei, and Yana rivers are not obvious, but the R_r_ is positive and the contribution rate is large. These three rivers are all Arctic rivers and are considered to be affected by glacial meltwater; that is, the $\delta_{c}$ in the basin is 71%, 72%, and 74%, respectively, so the discharge is mainly affected by climatic factors. The North Dvina River is also an Arctic river with few human activities and a positive value of R_r_ in the basin; therefore, R_r_ mainly indicates an increase in glacial meltwater and $\delta_{c}$ is 77%. In the Lena River Basin, although the contribution rate of precipitation is only 33%, the precipitation has a clear upward trend and passes the 99% significance test. It is also an Arctic river, the R_r_ is positive and the contribution rate is large, and the influence of glacial meltwater is also considered to be large, so the $\delta_{c}$ is 74%. The R_r_ of the Orinoco River is negative, indicating artificial water withdrawal. The Mississippi River Basin is less affected by glacial meltwater, and it is considered that R_r_ mainly means artificial water transfer, that is, the $\delta_{l}$ values are 26% and 38%. According to the analysis, seven river discharges with significant upward trends are mainly affected by climatic factors.

In the 12 basins where the discharge has a significant downward trend, the $\delta_{z}$ values in the four basins are greater than 50%. Among them, the precipitation in the Congo River Basin showed a significant downward trend, and the significance test confidence level reached 99.9%; thus, the decrease in discharge was mainly due to a significant decrease in precipitation. In the Sao Francisco River Basin, the precipitation did not change significantly, but the $\delta_{E_{0}}$ and the $\delta_{n}$ values is relatively high (19% and 37%). So we considered that the significant decrease in discharge is both affected by climatic factors and human activities. In the four basins, only the R_r_ value in the Fraser River Basin is positive due to the mountainous areas and few human activities in the basin. It is considered that R_r_ mainly indicates an increase in glacial meltwater, that is, the $\delta_{c}$ is 76%. The R_r_ values in the Congo River and the Sao Francisco River are negative, which suggest artificial water withdrawal, that is, the $\delta_{l}$ values are 35% and 42%, respectively. In the Yellow River Basin, the $\delta_{z}$ is 50%, and the R_r_ is negative, indicating human water withdrawal; thus, the $\delta_{l}$ is 50%, which shows that both climatic and human activity has an impact, but the $\delta_{n}$ is 41%; therefore, it is believed that the influence of human activities in this basin is dominant. The $\delta_{z}$ in the seven basins is less than 50%, of which the $\delta_{z}$ in Columbia River is 49% and the R_r_ is positive. Due to large amounts of water conservancy and hydropower projects in the basin, discharge is greatly disturbed by human activities, that is, R_r_ mainly means artificial water transfer. Additionally, the R_r_ values in the other six rivers are negative, which means artificial water withdrawal, that is, the $\delta_{c}$ of the seven rivers is consistent with the $\delta_{z}$. Based on the above analysis, in the 12 basins with a significant downward trend, the Fraser, Ganges, Congo and Sao Francisco River Basins are mainly affected by climatic factors, and the remaining 8 rivers are mainly affected by human activities.

In the 11 rivers with an insignificant upward trend, only the R_r_ in the five watersheds, such as the Magdalena River, is negative, which indicates artificial water withdrawal. The R_r_ in the Neva River is positive with a contribution rate of 3%. Because the basin mainly includes three large lakes and the human activities are less affected, the $\delta_{c}$ is considered to be 74%. Precipitation in the Magdalena and Neva rivers showed a significant upward trend, which passing 90% and 95% significant tests, respectively. The main factors affecting discharge changes in these two basins were climate change. So the discharge shows an insignificant upward trend that can be attributed to increased precipitation. The R_r_ values in the Ob, Pechora and Kolyma rivers are positive, and the three rivers are all Arctic rivers. The R_r_ is considered to be mainly affected by glacial meltwater; that is, the $\delta_{c}$ values in the three basins are 64%, 79%, and 71%. The R_r_ of the Yangtze and Nelson rivers are positive, and the contribution rate is 4%. Because of the large number of water conservancy projects in these two basins, the $\delta_{l}$ in the two basins were 31% and 39%. According to the above analysis, the discharge of the Zambezi and the Colorado River Basins is mainly affected by human activities, and the remaining 9 rivers are mainly affected by climatic factors.

In the 10 watersheds with an insignificant downward trend, the R_r_ values in the Rhine, Pearl, Mekong and Niger River Basins are negative and indicate artificial water withdrawal and the values in the four basins are 27%, 34%, 39%, and 55%, respectively. The precipitation in the Niger River Basin shows a significant downward trend, passing a 90% significance test. It is believed that the cause of the discharge changes includes both precipitation and human activities, but human activities have a greater impact. The R_r_ values in the Yukon and the Mackenzie River Basin are positive, and the two rivers are all Arctic rivers. The human activities in these basins are less, and the R_r_ mainly indicates an increase in glacial meltwater and the $\delta_{c}$ values in the two basins are 70% and 69%, respectively. The R_r_ in the Amazon, St. Lawrence, Danube and Amur River Basins are positive with contribution rates of 6%, 3%, 9%, and 7%, respectively, indicating that the impact of R_r_ on discharge changes is relatively small and the four basins are less affected by glacial meltwater, so R_r_ mainly indicates artificial water transfer and the $\delta_{l}$ in the four basins are 26%, 30%, 35%, and 42%. According to the above analysis, only the Niger River discharge is greatly affected by human activities, and the remaining nine rivers are mainly affected by climatic factors.

**Figure 7:**
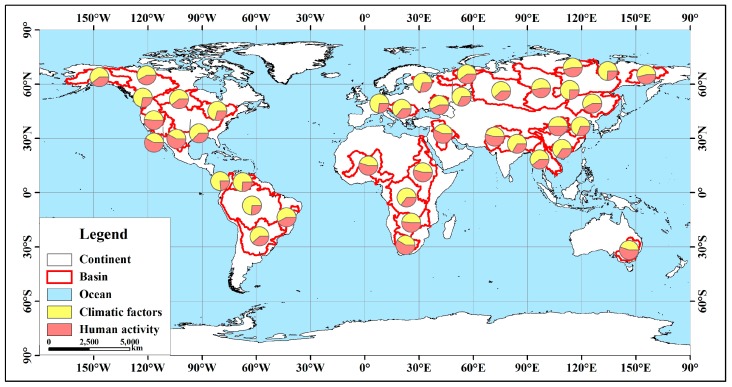
Distribution of the influencing factors of the discharge variation in each basin.

Based on the above analysis, 29 river basin discharge changes are mainly affected by climatic factors, and 11 river basins are mainly affected by human activities, including the Yellow, Columbia, Nile, Indus, Murray-Darling, Rio Grande, Orange, Zambezi, Colorado, Niger, and the Two River Basin. The distribution of factors affecting the discharge variation in each basin is shown in Figure 7.

These results are generally consistent with previous studies. For the Yellow, Colorado, Murray-Darling, Orange and Columbia River, the contribution rate of human activities in this study is between 50% and 60%. It is considered that both climatic factors and human activities have effects, which is similar to the result of some studies [17,18,20,21,22,23]. But the result of the Murray-Darling River is different from that of the study by Potter and Chiew [20]. This may be due to different research periods. For the Arctic rivers (except the Yana River), the sum of the contribution rates of precipitation and evapotranspiration is greater than 50%, indicating that climatic factors are still the main influencing factors. This is generally similar to the results studied by Brabets et al. [12] and Adam et al. [15]. The influencing factors include snowmelt, permafrost degradation, and increased fire frequency in their research. For the Yenisei and Lena River, the contribution rate of human water transfer and glacial melting in this study is relatively large (19% and 25%), which is generally similar to the results studied by Berezovskaya et al. [9,11] and Stuefer et al. [10]. Since the discharge directed into the ocean is the link between the terrestrial water cycle and the ocean water cycle, Su et al. believed that discharge changes are also related to ocean signals such as El Niño–Southern Oscillation (ENSO), Arctic Oscillation (AO), North Atlantic Oscillation (NAO) and Pacific Decadal Oscillation (PDO) [52].

### 4.2. Impact of Climate Zone on River Discharge

In the Mississippi, Yangtze, Pearl and the Parana River Basins, the main climate type is a subtropical monsoon humid climate, and the discharge changes are dominated by climatic factors. All basins are located between 20°–40° in the north and south latitudes, except for the insignificant downward trend of the Pearl River discharge, the other three basins all have an upward trend, therefore, the discharge in the basin in the subtropical monsoon humid climate zone mainly has an upward trend. The Congo and the Amazon River Basin include the tropical rainforest climate, and the discharge changes are dominated by climatic factors. The discharge is declining, and the confidence level in the Congo River Basin is 99.9%. It is considered that the discharge in the tropical rainforest climate zone mainly has a downward trend. The Magdalena, Orinoco, Sao Francisco and the Zambezi River Basins all contain a large proportion of the tropical savanna climate, except for the downward trend of the discharge in the Sao Francisco River Basin, the other basins showed an upward trend, and in the Orinoco River, the confidence level reached 99%. As mentioned above, the conditions of the underlying surface in the Sao Francisco River Basin are relatively large, so the discharge in the tropical savanna climate zone mainly has an upward trend. The Alpine climate accounts for a large proportion in the Columbia and Fraser River Basins, and the discharge changes in both basins are significantly declining. The discharge changes in the Fraser River Basin are mainly affected by climatic factors. Both climate factors and human activities impact the Columbia River Basin, so the change in discharge in the Alpine climatic zone mainly shows a downward trend, but the confidence is low.

The tropical monsoon climate is the main climate type in the Ganges and the Mekong River Basin, and the discharge is mainly affected by climatic factors and is decreasing, therefore, the discharge in the tropical monsoon climate zone mainly shows a downward trend. The temperate monsoon climate in the Amur River Basin is the main climate type and the $\delta_{c}$ in the basin is 58%, but the $\delta_{n}$ is 35%. The discharge in this basin is decreasing but is not significant, so the discharge changes in the temperate monsoon climate are not obvious. In the 11 river basins, namely, the Yukon, Mackenzie, St. Lawrence, Nelson, Northern Dvina, Danube, Don, Pechora, Neva, Ob and Kolyma River Basin, most of the climate types are the temperate continental climate, and the discharge changes are mainly affected by climatic factors; however, only the discharge in the Northern Dvina River Basin shows a significant trend. Due to the wide distribution of temperate continental climatic zones, the regional differences are large, so the trend is insignificant in the temperate continental climate zone. In the Rhine River Basin, the temperate marine climate is the main climate type, the change in discharge is dominated by climatic factors, and the discharge trend is not obvious. Thus, the discharge in the temperate maritime climate zone is considered basically stable.

Although few studies have focused on the impact of climatic zones on runoff, some studies have been summarized by geographic location and can also support the findings of this paper. For example, runoff in the subtropical humid monsoon and savanna climate zones predominantly showed upward trends in this study, and eight rivers (the Mississippi, Yangtze, Pearl, Parana, Magdalena, Orinoco, Sao Francisco, and Zambezi River) mainly contain these two climatic zones. The studies (Dai et al. [25] and Milly et al. [53]) concluded that the runoff near the Gulf of Mexico, southern South America, and southeastern Africa showed upward trends. In this study, runoff in the tropical rainforest and the tropical monsoon climate zones predominantly showed downward trends, and four rivers (the Congo, Amazon, Ganges, and Mekong River) mainly contain these two climatic zones. In the conclusion studied by Milly et al. [53] and Gerten et al. [54], the runoff in Sub-Saharan Africa and Central /South Asia showed downward trends, which is generally similar to the results in this study.

### 4.3. Uncertainty

During the data selection, we first considered the data abundance at most of the sites and the lack of data at other sites. Then we considered the impact of climate change and human activities and chose 1960 as the base year. The data series are mainly measured data, and the missing data is obtained by data extension and interpolation from other data sources. This process may cause data errors, but from the result analysis, the general trend of change is basically consistent with the previous study [15,16,18,20,22,24,25], so the error caused by the data is negligible.

In the analysis of the attribution of the changes in river discharge, we developed the analysis method of the discharge variation attribution based on the Budyko hypothesis to adapt for large-scale basins. The influence factors of human activities and glacial meltwater were added into the formula represented by R_r_. In the analysis of the effects of R_r_, qualitative identification is currently only based on the geographical location of the basin and the development of human society. For example, the Arctic basin was less affected by human activities, and the R_r_ in these basins represents the influence of glacial meltwater. If there are many water conservancy and hydropower projects in the basin and the glacier has less impact, then the R_r_ represents artificial water transfer. Due to the large scale of the basin, the error is relatively small. In future study, more data will be considered for in-depth analysis.

## 5. Conclusions

By analyzing the trends and distribution characteristics and the attribution of the discharges directed into the ocean in the 40 major global rivers, we conclude the following:

(1) At present, the discharges of the major global rivers directed toward the oceans more preferentially showed downward trends and fewer upward trends. The downward trends accounted for 57.5% and the upward trends accounted for 42.5% of the total. In North America and Africa, approximately two-thirds of the rivers showed downward trends. In Europe, 80% of the rivers showed upward trends. In Asia and South America, the number of rivers that showed upward trends and downward trends was approximately equal. In Oceania, the Murray-Darling River is the major river, and it showed a significant downward trend. Regarding the segmentation trend, the North American rivers mainly show the trend of “wet-dry” with mutation points around the 1980s. The five rivers in Africa all showed the trend of “wet-dry” with a mutation time in approximately the 1970s. The mutations in different rivers in South America were different. The mutation time in the European and Asian rivers was mainly in the 1980s and 1990s.

(2) Climatic factors were the main influencing factors for the change in the discharge of major global rivers directed into the ocean, and only approximately 25% of river discharge was greatly affected by human activities. From the perspective of the climate zone, the river discharge in the subtropical monsoon humid climate and tropical savanna climate zone mainly exhibited an upward trend. In the tropical rainforest climate and the tropical monsoon climate zone, river discharge mainly demonstrated a downward trend. The change in the river discharge in the temperate monsoon climate, the temperate continental climate, and the temperate marine climate were relatively stable. From the perspective of latitude, the discharge in the mid-high latitudes mainly showed an upward trend, and the discharge in the mid-low latitudes with the influence of human activities mainly showed a downward trend. The runoff coefficient also decreased.

## Figures and Tables

**Table 1:** River information and data sources.

No.	Continent	River	Station	Area (km^2^)	Data Source
1	NA	Yukon	PILOT	922963	USGS, GRDC, [33]
2	NA	Colorado	ABOVE MORELOS DAM	805231	USGS
3	NA	Mackenzie	ARCTIC RED RIVER	1831060	GRDC, [33]
4	NA	Mississippi	VICKSBURG	3287560	GRDC, USGS
5	NA	St. Lawrence	LASALLE	1109840	HYDAT
6	NA	Columbia	BEAVER ARMY TERMINAL	1099310	GRDC
7	NA	Rio Grande	MATAMOROS	841399	GRDC
8	NA	Nelson	LONG SPRUCE GENERATING	1232640	GRDC
9	NA	Fraser	HOPE	188653	GRDC
10	OA	Murray-Darling	LOCK 1 DOWNSTREAM	1047620	ABM, GRDC, [33]
11	AF	Orange	VIOOLSDRIF	975391	GRDC
12	AF	Congo	KINSHASA	3761150	GRDC
13	AF	Nile	EL EKHSASE	3314920	GRDC, [33,34]
14	AF	Niger	LOKOJA	2673950	GRDC, [33,35]
15	AF	Zambezi	MATUNDO-CAIS & SHIRE	2076310	GRDC, [33]
16	SA	Parana	TIMBUES	3525050	GRDC, [36]
17	SA	Amazon	OBIDOS	6866290	GRDC, SO HYBAM
18	SA	Magdalena	CALAMAR	261432	GRDC, [37]
19	SA	Orinoco	PUENTE ANGOSTURA	985093	GRDC, [38]
20	SA	Sao Francisco	TRAIPU	649467	GRDC
21	EU	Danube	CEATAL	788476	GRDC
22	EU	Rhine	LOBITH	185908	GRDC
23	EU	Don	RAZDORSKAYA	438258	GRDC, [39]
24	EU	Northern Dvina	UST-PINEGA	333191	GRDC
25	EU	Pechora	OKSINO	311687	GRDC
26	EU	Neva	NOVOSARATOVKA	278039	GRDC
27	AS	Ob	SALEKHARD	3227630	GRDC, [14]
28	AS	Yellow	LIJIN	795044	CHINA WATER RESOURCES BULLETIN
29	AS	Lena	KYUSYUR (KUSUR)	2466930	GRDC
30	AS	Yenisei	IGARKA	2866740	GRDC
31	AS	Yangtze	DATONG	1782720	CHINA WATER RESOURCES BULLETIN
32	AS	Kolyma	KOLYMSKAYA	596116	GRDC, [14]
33	AS	Amur	BOGORODSKOYE	2161190	GRDC, [40]
34	AS	Indus	KOTRI	1500850	GRDC, [33,41]
35	AS	Ganges-Brahmaputra	PAKSEY, BAHADURABAD	1579040	GRDC, [33,42,43,44]
36	AS	Mekong	MY THUAN, VAM CONG	937943	GRDC, [33,45]
37	AS	Tigris and Euphrates	HINDIYA, BAGHDAD	1278420	GRDC, [46,47,48]
38	AS	Yana	UBILEYNAYA	231156	GRDC
39	AS	Olenyok	7.5 KM D/S OF MOUTH	218112	GRDC
40	AS	Pearl	GAOYAO, SHIJIAO, BOLUO	448701	CHINA WATER RESOURCES BULLETIN

**Table 2:** Results of the linear tendency estimation and M-K tests.

No.	Continent	River	Linear Trend	Linear Sig. ^a^	Z	M-K Sig. ^b^	Trend ^c^
1	NA	Yukon	−0.182		−0.89		−−
2	NA	Colorado	0.003		0.52		+
3	NA	Mackenzie	−0.054		0.49		−
4	NA	Mississippi	2.522	*	2.31	**	++
5	NA	St. Lawrence	0.067		−0.28		−
6	NA	Columbia	−0.595	*	−1.77	*	−−
7	NA	Rio Grande	−0.013	*	−2.52	**	−−
8	NA	Nelson	0.121		0.39		+
9	NA	Fraser	−0.283	*	−2.62	***	−−
10	OA	Murray-Darling	−0.116	*	−2.78	***	−−
11	AF	Orange	−0.076	*	−2.05	**	−−
12	AF	Congo	−4.791	*	−2.75	***	−−
13	AF	Nile	−0.369	*	−2.18	**	−−
14	AF	Niger	−0.055		−0.37		−
15	AF	Zambezi	−0.085		0.45		+
16	SA	Parana	3.495	*	1.56		+
17	SA	Amazon	−1.676		−0.63		−
18	SA	Magdalena	0.444		0.76		+
19	SA	Orinoco	2.283	*	2.5	**	++
20	SA	Sao Francisco	−0.412	*	−1.87	*	−−
21	EU	Danube	−0.015		−0.24		−
22	EU	Rhine	−0.036		−0.21		−
23	EU	Don	−0.006		0.31		+
24	EU	Northern Dvina	0.311	*	1.98	**	++
25	EU	Pechora	0.223		0.96		+
26	EU	Neva	0.104		1.23		+
27	AS	Ob	0.273		−0.16		+
28	AS	Yellow	−0.832	*	−5.34	****	−−
29	AS	Lena	1.402	*	2.18	**	++
30	AS	Yenisei	1.768	*	3.36	****	++
31	AS	Yangtze	0.534		0.19		+
32	AS	Kolyma	0.308	*	1.5		+
33	AS	Amur	−1.016	*	−1.57		−
34	AS	Indus	−0.433	*	−2.21	**	−
35	AS	Ganges-Brahmaputra	−2.474	*	−2.1	**	−−
36	AS	Mekong	−0.382		−0.89		−
37	AS	Tigris and Euphrates	−0.583	*	−3.4	****	−−
38	AS	Yana	0.191	*	2.23	**	++
39	AS	Olenyok	0.302	*	2.91	***	++
40	AS	Pearl	−0.096		−0.63		−

^a^ * indicates a clear trend; spaces indicate that the trend is not obvious. ^b^ * represents a significance level of α = 0.1, ** α = 0.05, *** α = 0.01, and **** α = 0.001. ^c^ ++ represents a significant upward trend, −− represents a significant downward trend, + represents an upward trend that is not significant, and − represents a downward trend that is not significant.

**Table 3:** Mutation and segmentation trends of the river discharge changes.

River	Cumulative Anomaly	M-K Mutation Point	Segmentation Trends
Yukon	1967,1978,1995	1965,2000	1960–1967 wet; 1967–2010 dry
Colorado	1978,1987	1977,2006	1960–1978 dry; 1978–1987 wet; 1987–2010 dry
Mackenzie	2004	1962,2007	1960–2004 dry; 2004–2010 wet
Mississippi	1971	1971	1960–1971 dry; 1971–2010 wet
St. Lawrence	1971,1998	1966,1999	1960–1971 dry; 1971–1998 wet; 1998–2010 dry
Columbia	1986,1994,1999	1986,1995,1998	1960–1986 wet; 1986–2010 dry
Rio Grande	1981	1993	1960–1981 wet; 1981–2010 dry
Nelson	1975	1973	1960–1975 wet; 1975–2010 dry
Fraser	1976	1977	1960–1976 wet; 1976–2010 dry
Murray-Darling	1988,1996	2004	1960–1988 dry; 1988–1996 wet; 1996–2010 dry
Orange	1978	1979	1960–1978 wet; 1978–2010 dry
Congo	1970	1967	1960–1970 wet; 1970–2010 dry
Nile	1978	1970	1960–1978 wet; 1978–2010 dry
Niger	1970	1970	1960–1970 wet; 1970–2010 dry
Zambezi	1981	1981	1960–1981 wet; 1981–2010 dry
Parana	1981,1998	1970	1960–1981 dry; 1981–1998 wet; 1998–2010 dry
Amazon	1970,1978	1970,1990	1960–1970 dry; 1970–1978 wet; 1978–2010 dry
Magdalena	2005	1970,2007	1960–2005 dry; 2005–2010 wet
Orinoco	1997	1980,1998	1960–1997 dry; 1997–2010 wet
Sao Francisco	1986	1983	1960–1986 wet; 1986–2010 dry
Danube	1981,1995	1981	1960–1981 wet; 1981–2010 dry
Rhine	1970,1977,1988	1971,1977,1989,2003	1960–1970 wet; 1970–1977 dry; 1977–1988 wet; 1988–2010 dry
Don	1993	1992	1960–1993 dry; 1993–2010 wet
Northern Dvina	1982	1982	1960–1982 dry; 1982–2010 wet
Pechora	1990	1995	1960–1990 dry; 1990–2010 wet
Neva	1980,1995	1980,1996	1960–1980 dry; 1980–1995 wet; 1995–2010 dry
Ob	1968,1987	1969,1989	1960–1968 dry; 1968–1987 wet; 1987–2010 dry
Yellow	1985	1981	1960–1985 wet; 1985–2010 dry
Lena	2003	2005	1960–2003 dry; 2003–2010 wet
Yenisei	1987	1991	1960–1987 dry; 1987–2010 wet
Yangtze	1979	1979	1960–1979 dry; 1979–2010 wet
Kolyma	1995	1996	1960–1995 dry; 1995–2010 wet
Amur	1980,1999	1980,2000	1960–1980 dry; 1980–1999 wet; 1999–2010 dry
Indus	1999	2000	1960–1999 wet; 1999–2010 dry
Ganges-Brahmaputra	1990	1991	1960–1990 wet; 1990–2010 dry
Mekong	1973,1993	1973	1960–1973 wet;1973–1993 dry;1993–2010 wet
Tigris and Euphrates	1989	1990	1960–1989 wet; 1989–2010 dry
Yana	1995	1996	1960–1995 dry; 1995–2010 wet
Olenyok	1988	1986	1960–1988 dry; 1988–2010 wet
Pearl	1983,1992,2002	1989,2003	1960–1983 wet; 1983–1992 dry;1992–2002 wet; 2002–2010 dry

**Table 4:** Underlying surface coefficient and elastic coefficient and contribution rate of each influencing factor of each basin.

River	Z (P)	P Sig. ^1^	*n*	$\varepsilon_{P}$	$\varepsilon_{R_{r}}$	$\varepsilon_{E_{0}}$	$\varepsilon_{n}$	$\delta_{P}$	$\delta_{R_{r}}$	$\delta_{E_{0}}$	$\delta_{n}$	$\delta_{z}$	$\delta_{c}$	$\delta_{l}$
Orinoco	1.59		1.1	1.73	−0.14	−0.60	−0.68	55	4	19	22	74	74	26
Northern Dvina	1.72	*	1.3	1.31	0.25	−0.56	−0.63	48	9	20	23	68	77	23
Mississippi	1.41		1.2	1.86	0.05	−0.91	−1.64	42	1	20	37	62	62	38
Olenyok	0.44		1.0	0.94	0.43	−0.36	−0.69	39	18	15	29	54	71	29
Yenisei	1.09		1.2	0.95	0.49	−0.44	−0.73	36	19	17	28	53	72	28
Lena	3.09	***	1.2	0.77	0.59	−0.36	−0.60	33	25	16	26	49	74	26
Yana	1.06		1.2	0.65	0.66	−0.30	−0.56	30	30	14	26	44	74	26
Fraser	−0.63		1.0	1.33	0.10	−0.43	−0.60	54	4	18	24	72	76	24
Ganges	−0.47		0.7	1.36	0.00	−0.36	−0.86	53	0	14	33	67	67	33
Congo	−3.48	****	1.2	2.59	−0.46	−1.12	−1.58	45	8	20	27	65	65	35
Sao Francisco	0.03		1.2	2.69	−0.36	−1.33	−2.61	38	5	19	37	57	57	42
Yellow	−0.77		1.1	3.87	−1.06	−1.81	−4.61	34	9	16	41	50	50	50
Columbia	−0.79		1.0	0.96	0.46	−0.42	−1.01	34	16	15	35	49	49	51
Nile	−1.43		0.8	9.99	−5.16	−3.83	−11.22	33	17	13	37	46	46	54
Indus	1.14		0.8	4.33	−1.72	−1.60	−5.32	33	13	12	41	46	46	54
Murray-Darling	−0.03		1.0	23.26	−11.57	−10.69	−30.36	31	15	14	40	45	45	55
Two Rivers	−1.71	*	0.5	4.24	−2.10	−1.14	−5.02	34	17	9	40	43	43	57
Rio Grande	0.60		0.8	116.8	−68.61	−47.18	−159.5	30	18	12	41	42	42	58
Orange	1.15		0.7	17.50	−9.84	−6.66	−24.71	30	17	11	42	41	41	59
Magdalena	1.84	*	1.3	1.86	−0.17	−0.69	−0.63	56	5	21	19	76	76	24
Neva	2.44	**	1.2	1.56	0.10	−0.65	−0.81	50	3	21	26	71	74	26
Yangtze	−0.47		1.0	1.42	0.11	−0.53	−0.78	50	4	19	28	69	69	31
Ob	1.51		1.1	1.67	0.08	−0.76	−1.41	43	2	19	36	62	64	36
Parana	1.32		1.2	2.46	−0.32	−1.15	−1.93	42	5	20	33	62	62	38
Nelson	0.71		1.5	1.80	0.20	−1.01	−1.64	39	4	22	35	61	61	39
Pechora	1.06		0.9	0.90	0.37	−0.27	−0.41	46	19	14	21	60	79	21
Don	0.69		1.3	3.20	−0.51	−1.69	−3.22	37	6	20	37	57	57	43
Kolyma	0.62		1.0	0.94	0.42	−0.36	−0.70	39	17	15	29	54	71	29
Zambezi	0.02		0.9	7.42	−3.34	−3.08	−7.80	34	15	14	36	48	48	52
Colorado	0.36		1.0	11.97	−5.33	−5.64	−18.80	29	13	14	45	43	43	57
Amazon	0.18		1.3	1.40	0.15	−0.55	−0.54	53	6	21	20	74	74	26
Rhine	0.45		1.1	1.72	−0.08	−0.65	−0.81	53	2	20	25	73	73	27
St. Lawrence	1.40		1.6	1.88	0.10	−0.99	−1.10	46	3	24	27	70	70	30
Pearl	−0.36		1.0	2.38	−0.61	−0.77	−1.02	50	13	16	21	66	66	34
Danube	0.55		1.5	1.43	0.30	−0.73	−0.91	43	9	22	27	65	65	35
Yukon	−0.76		0.9	1.12	0.25	−0.37	−0.74	45	10	15	30	60	70	30
Mekong	0.34		1.1	3.55	−1.19	−1.36	−2.05	44	15	17	25	61	61	39
Mackenzie	0.06		1.2	1.21	0.33	−0.54	−0.94	40	11	18	31	58	69	31
Amur	−1.15		1.2	1.40	0.26	-0.66	−1.24	39	7	19	35	58	58	42
Niger	−1.71	*	0.6	3.93	−1.68	−1.25	−4.61	34	15	11	40	45	45	55

^1^ * indicates a confidence level of 90%; ** indicates a confidence level of 95%; *** indicates a confidence level of 99%; **** indicates a confidence level of 99.9%.
